# DSC Analysis of the Effect of Cold Deformation on the Precipitation Kinetics of a Binary Cu-Sc Alloy

**DOI:** 10.3390/ma16093462

**Published:** 2023-04-29

**Authors:** Ramona Henle, Julia Dölling, Ulrich Prahl, Gerrit Nandi, Andreas Zilly

**Affiliations:** 1 Faculty of Technology, Cooperative State University Heidenheim, Marienstraße 20, 89518 Heidenheim an der Brenz, Germany; gerrit.nandi@dhbw-heidenheim.de; 2 Faculty of Technology, Cooperative State University Stuttgart, Lerchenstraße 1, 70197 Stuttgart, Germany; julia.doelling@dhbw-stuttgart.de (J.D.); andreas.zilly@dhbw-stuttgart.de (A.Z.); 3 Institute of Metal Forming, Technische Universität Bergakademie Freiberg, Bernhard-von-Cotta Straße 4, 09599 Freiberg, Germany; ulrich.prahl@imf.tu-freiberg.de

**Keywords:** copper–scandium CuSc, copper alloy, differential scanning calorimetry DSC, precipitation kinetics, cold-working, cold-rolling, activation energy, Kissinger method

## Abstract

The present study aimed to investigate the effect of cold deformation on the precipitation kinetics of a binary CuSc alloy containing 0.4 wt.% scandium using the experimental analysis method of differential scanning calorimetry (DSC). Non-deformed and 75% cross-section-reduced cold-rolled supersaturated specimens were tested in non-isothermal DSC runs at up to five different heating rates. The DSC results showed that cold rolling significantly accelerated the precipitation process in the binary alloy, leading to a decrease in the initial and peak temperatures of the exothermic reactions. The activation energies calculated with the Kissinger method indicated that the precipitation activation energy decreased with increasing cold deformation. The findings of this study provide worthy implications to further optimize the processing of Cu-Sc alloys with improved mechanical properties.

## 1. Introduction

Microalloyed copper alloys are used in many areas of industry, exhibiting high electrical and thermal conductivity as well as increased mechanical strength [1]. Therefore, they are frequently used for signal cables, connectors, or welding electrodes, particularly in the electrical industry. There are several hardening mechanisms for copper, which is very soft in its pure state [2], that can bring about the desired properties. One of the most important in this context is precipitation hardening, which allows both, the otherwise conflicting mechanical strength of an alloy and the electrical conductivity to be increased by optimizing the microstructural properties during specific heat treatments [2,3,4,5].

However, a conflict arises from the fact that precipitates can act as scattering centers for current flow. The smaller and more numerous the precipitates are, the more they impede the flow of electric current and reduce the conductivity of the material [6]. To balance the trade-off between mechanical strength and electrical conductivity, it is essential to carefully control the size, distribution, and morphology of precipitates. This can be achieved through a combination of processing conditions such as heating rate, aging temperature, and prior cold working, as well as the alloy composition [2,3,6,7].

Recently published studies by Franczak et al. [8] and Dölling et al. [9] have shown that a combination of copper and scandium has potential in terms of electrical conductivity and mechanical strength, in addition to having advantages in terms of recrystallization behavior and grain refinement [9,10,11]. In particular, cold working prior to hardening of the precipitates increases the density of dislocations and strain-induced defects in the material, creating more nucleation sites for fine precipitates to form.

**Figure 1:**
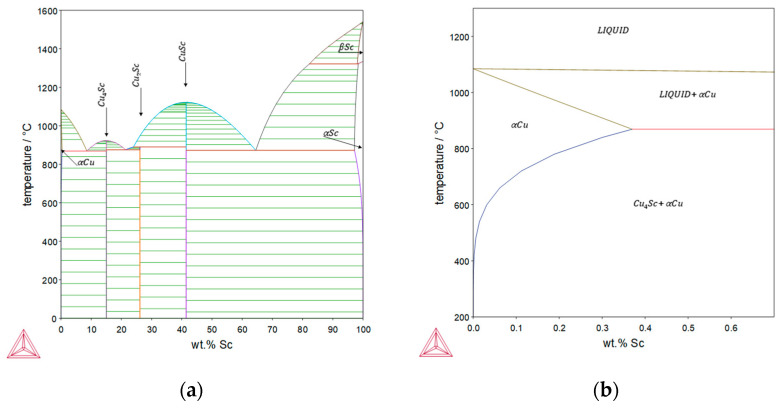
Phase diagram of the binary Cu-Sc system calculated with the Thermo-Calc SGTE database (2022a): (**a**) overall; (**b**) magnification of the copper-rich area [9].

Figure 1 shows the phase diagram of binary Cu-Sc alloy. The maximum solubility of scandium in copper is reported to be 0.35 wt.% at 865 °C [12,13,14] and decreases with decreasing temperature, leading to the possibility of precipitation formation in binary Cu-Sc alloy [15].

Hao et al. investigated the precipitation behavior and hardening effects of a highly deformed and cryorolled CuSc0.4 wt.%. The precipitation reaction started initially from the supersaturated solid solution, forming Sc-rich atomic clusters. Subsequently, these formed coherent lamellar precipitates and became increasingly detached from the matrix structure to become tetragonally-oriented lamellar Cu_4_Sc precipitates [11,16]. The precipitation strengthening that occurs at this point is described by Hao et al. as a combination of coherent strengthening with significant matrix distortion and presence of the incoherent Orowan bypass mechanism at larger precipitate sizes [11,17].

To optimize precipitation treatments for materials with desired properties, differential scanning calorimetry (DSC) can be used to develop a deeper understanding of the underlying precipitation mechanisms. DSC analysis is a common thermal analysis technique that measures the heat flow into or out of a material as a function of temperature and time. Therefore, it can detect reactions within the microstructure as exothermic (precipitation and recrystallization) or endothermic (dissolution) processes [18,19]. This analysis provides valuable information about the thermal behavior of a material, such as the melting point, crystallization kinetics and phase transitions [18,20,21,22]. In the context of precipitation kinetics, DSC analysis can be used to study the evolution of precipitates in a material during aging or heating. By monitoring the heat flux, DSC provides information on precipitation kinetics such as the nucleation and growth rate of precipitates, as well as the temperature dependence of precipitation reactions [21,23,24].

An important parameter characterizing the kinetics of a reaction is the activation energy, which can be obtained from DSC data using one of several available methods, including the Kissinger [25,26], Ozawa [27,28], and Boswell [29] methods. This can be used to predict the reaction behavior at different temperatures, such as the time required to complete the reaction to a certain degree, or to predict the temperature at which the reaction will occur. The Kissinger method is often the preferred approach for determining the activation energy of precipitation reactions using DSC, as it assumes a single reaction mechanism and only requires a single DSC measurement at each heating rate, making it a simple and convenient approach that can provide valuable information for materials science and engineering applications [23,30].

This study aims to determine the activation energy required for the precipitation reaction in an undeformed alloy and a 75% cold-worked CuSc0.4 alloy.

## 2. Materials and Methods

The composition of the alloy was analyzed using a specially calibrated optical emission spark spectrometer (Spectrotest, SPECTRO Analytical Instruments GmbH, Kleve, Germany) and determined to be Cu with a content of 0.40 wt.% Sc. From this, an ingot was cast on a VC400 casting machine (Indutherm Blue Power Casting Systems, Walzbachtal, Germany) utilizing the raw materials Cu-OFE and CuSc23 as a master alloy. Melting was performed in a boron nitride-covered graphite crucible up to 1300 °C, which along with the casting process was performed under vacuum conditions. After pouring in a graphite mold (heated at 250 °C), the 5 mm thick bar was solution annealed at 870 °C for 120 min in a preheated furnace (ME65/13, Helmut ROHDE GmbH, Prutting, Germany) and then quenched to room temperature in circulated water. The as-quenched plate was divided and one part was longitudinally cold rolled on a duo-roll stand (Bühler, Pforzheim, Germany) with 110 mm diameter rolls driven with a speed of 27 min^−1^.

Differential scanning calorimetry (DSC) analysis of Cu-Sc alloys was performed using a Netzsch STA 449 C. The calorimeter calibration was performed thermally with Al_2_O_3_ crucibles by melting In, CsCl, Ag, and Au to obtain a baseline. The mass of the samples ranged from 40 to 202 mg. Several measurements of the same heating rate with higher sample mass resulted in identical curves, though with an increased signal-to-noise ratio (SNR). During the heating process, a protective argon atmosphere (20 mL/min) and purge gas (30 mL/min) were utilized, and an empty crucible was used as a reference. The precipitation experiments were carried out using continuous heating rates (5, 10, 15, 20, and 40 K/min) with a temperature ranging from room temperature (RT) to 750 °C. Data output was performed with an accuracy of 0.5 °C; the error for the DSC measurements as compared to the calibration measurements is shown in Table A1 of Appendix A.

The raw data from the DSC measurements were smoothed using a locally-weighted linear regression with the second-degree polynomial (LOESS) function in MATLAB with a span of 5%. Then, the second derivative of the function was calculated in order to determine the initial and final temperatures of the exothermic peak (*T_i_* and *T_f_*), which can be seen as inflection points in the curve. Estimation of the baseline of each curve was performed by spline interpolation of the smoothed DSC signal, with the maximum difference value between the smoothed DSC signal and the baseline indicating the peak temperature of the exothermic reaction (*T_p_*).

The activation energy of the precipitation of the Cu_4_Sc phase was calculated based on the dependence of previously determined *T_p_* temperatures on the heating rates using the Kissinger equation [25,26] provided by

(1) $$\ln\left(\frac{V}{T_{p}{}^{2}}\right)=C-\frac{E}{RT_{p}}$$

where *V* is the heating rate, *T_p_* is the peak temperature, *C* is a constant, *E* is the apparent activation energy, and *R* is the molar gas constant. By plotting ln(*V*/*T_p_*^2^) as a function of 1/*T_p_* and fitting a linear regression line *y* to the data, the activation energy *E* can be calculated from the slope of the line using the relationship

(2) E=−slope×R.

For microscopic analysis, one of the non-deformed CuSc0.4 specimens were heated using an identical heating process with a heating rate of 10 K/min up to 610 °C and cooled with the same temperature gradient. Thus, the microscopic evolution is directly comparable to measured exothermic peaks during the precipitation reaction in the DSC. Microstructure characterizations were observed with a scanning electron microscope (SEM) (Gemini Sigma VP with the used NTS BSD (Carl Zeiss Microscopy Deutschland GmbH, Oberkochen, Germany)) operating at 12 kV and Bruker XFlash 6|30 detector (Bruker Nano GmbH, Berlin, Germany).

## 3. Results and Discussion

The following chapter presents and discusses the results obtained from investigating the effect of cold deformation on the precipitation kinetics of a binary Cu-Sc alloy. This chapter is divided into three sections: DSC analysis, calculation of the activation energy, and microscopic analysis. Each section provides a comprehensive examination of the experimental findings and their implications for understanding the alloy’s precipitation behavior with and without prior cold deformation.

### 3.1. DSC Analysis

During the DSC analysis, precipitation reactions with a clearly visible exothermic peak appeared. Depending on the chosen heating rate, the location of this peak was slightly different.

**Figure 2:**
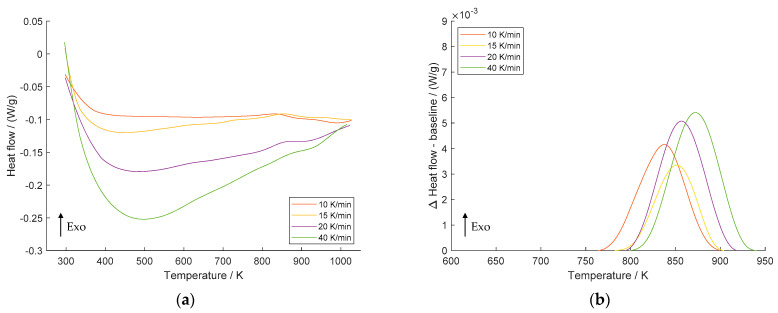
DSC scans of non-deformed CuSc0.4 alloy at heating rates of 10 K/min, 15 K/min, 20 K/min, and 40 K/min (**a**); difference between heat flow and the baseline of the precipitation reaction (**b**).

The DSC scans at different heating rates (10, 15, 20, and 40 K/min) for the non-deformed CuSc0.4 specimen (Figure 2a) all show an exothermic peak (Exo) between 760 K and 950 K. A closely related study by Dölling et al. [31] showed highly comparable curves for a non-deformed CuSc0.3 alloy at a 10 K/min heating rate with a peak temperature of 842.1 K. Furthermore, there was no recrystallization detected for non-formed Cu-Sc specimens, resulting in only a single peak indicating the precipitation reaction.

**Figure A1:**
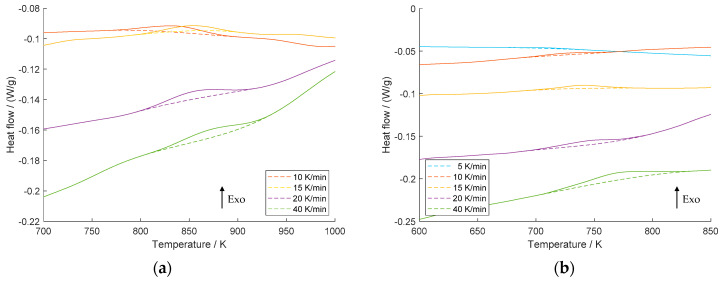
Overview of peak areas of non-deformed (**a**) and 75% cross-section-reduced (**b**) CuSc0.4, with DSC signal (straight lines) and interpolated baselines (dashed lines).

The difference between the smoothed DSC signal and the baseline and spline interpolations is displayed in Figure 2b. Obviously, the maximum temperature of the Cu_4_Sc precipitation shifts to higher values as the heating rate increases, implying that the precipitation reaction is thermally activated. Similar findings have been reported for recrystallization in cold-rolled pure copper [32], Cr clustering in equal-channel angular pressing (ECAP) processed CuCrZr [1], and recrystallization phenomena in Cu–Ni–Si alloy processed by high-pressure torsion (HPT) [33]. Due to the baseline shape extracted from the experiment, the determination of the 20 K/min curve using spline interpolation resulted in an exaggeratedly large area for the peak. At this point, manual adjustment of the baseline calculation parameters was necessary. Despite this adjustment, the peak temperature *T_p_* remained unchanged, thereby keeping any potential impacts on the calculation of the activation energy unaffected. Comprehensive insights pertaining to the interpolated baselines are provided in Appendix A, Figure A1.

With an increase in the heating rate, the temperature range at which the precipitation reaction takes place is widened. In addition, the initial (*T_i_*), peak (*T_p_*), and final (*T_f_*) temperatures shift to higher values, which can be attributed to the kinetics of the reaction. Reactions and transitions, such as precipitation or recrystallization, need time to transform, resulting in a narrower time range at lower heating rates due to the longer duration. On the other hand, at higher heating rates the time required to complete the reaction may not be sufficient because of the limitations imposed by the kinetics. For this reason, the peak expands at higher temperatures and widens with increasing heating rates.

However, scans of the non-deformed specimen at a heating rate of 5 K/min did not show a significant peak, which is why it is not mentioned in this study.

**Figure 3:**
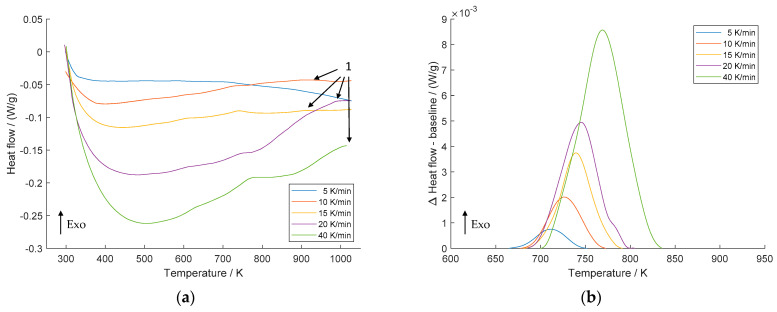
DSC scans of 75% cold-rolled CuSc0.4 alloy at heating rates of 5 K/min, 10 K/min, 15 K/min, 20 K/min, and 40 K/min (**a**); difference between heat flow and baseline of the precipitation reaction (**b**).

Figure 3 shows the DSC scans of the 75% cold-rolled Cu-Sc specimen at all heating rates (Figure 3a) and the differences between the precipitation peak curve and baseline (Figure 3b). Again, the maximum temperature shifts to higher temperatures at higher heating rates. However, in direct comparison to the non-deformed specimen, the precipitation reaction of the cold-rolled specimen starts about 100 K earlier (660–840 K).

In addition, the curves show the peak of the second exothermic reaction (indicated by the number 1 with arrows), which can be attributed to recrystallization of the microstructure. This investigation agrees with the findings of Dölling et al. [31] obtained with a directly comparable experimental setup and raw materials.

The obtained peak temperature values related to Cu_4_Sc precipitation as a function of heating rates are shown in Table 1. The temperature peaks ranged from 837.7 K to 872.3 K without prior cold deformation, while the peak temperatures of the 75% cold-rolled specimens ranged from 711.7 K to 769.0 K. It is obvious that the temperature peaks of the precipitation reaction decrease when increasing deformation is applied. This can be attributed to the fact that cold rolling introduces various lattice defects such as dislocations and vacancies into the material, which provide additional nucleation sites for precipitation, leading to an accelerated reaction. This effect was shown during isothermal aging experiments for comparable alloys (CuSc0.15 and CuSc0.3) and identical experimental processing conditions by Dölling et al. [9]. The mechanical and physical properties were analyzed under varying degrees of cold rolling during isothermal heat treatments, and the precipitation reactions occurred reproducibly earlier with higher degrees of cold rolling prior to aging at different temperatures. This phenomenon was evident due to the evolution of the material properties, namely, the concurrent increase in electrical conductivity and hardness. Furthermore, several previous investigations have shown that a high dislocation density significantly improves the kinetics of precipitation [5,34,35,36] and recrystallization [33,37,38].

### 3.2. Determination of Precipitation Activation Energies

The activation energy for scandium precipitation was calculated using the Kissinger method [25,26] using Formula 1. For this purpose, the values of the peak temperature *T_p_* and the corresponding heating rate *V* are used for the equation and then ln(*V/T_p_^2^*) is plotted versus 1000/K. Using Formula 2, the activation energy is derived from the slope of the linear fitting curve of the calculated points multiplied by the molar gas constant *R*.

**Figure 4:**
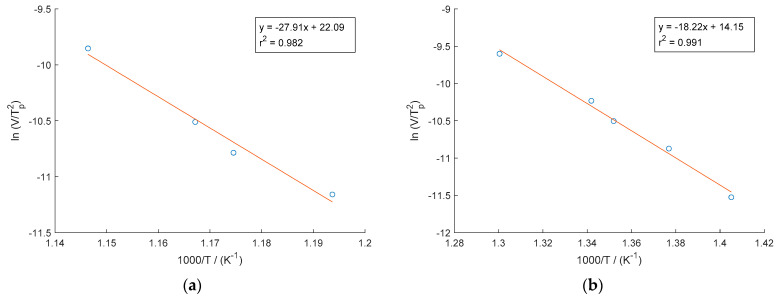
Kissinger plots of ln(*V/T_p_^2^*) against 1000/*T* of Cu_4_Sc precipitation in Cu-Sc alloy for non-deformed (**a**) and 75% cold-rolled (**b**) specimens.

Figure 4 shows the Kissinger plots of the non-deformed (Figure 4a) and cold-rolled specimens (Figure 4b) versus 1000/*T* for the precipitation reaction in the utilized CuSc0.4 alloys. All plots show straight lines, with high Pearson correlation coefficients of *r*^2^ = 0.991 for the undeformed specimen and *r*^2^ = 0.982 for the cold-rolled specimen. The activation energies were calculated from their slopes, and are listed in Table 2. The mean values of the activation energy for precipitation of the Cu_4_Sc phase are 232.02 kJ/mol and 151.51 kJ/mol, respectively.

However, temperature errors can cause inaccuracies in determination of the peak temperature during DSC measurements, potentially having a significant influence on the calculated activation energy. An error of just a few degrees between high and low heating rates can lead to an activation energy error of 10–20% [30]. Thus, careful temperature calibration and control to minimize such errors are crucial in DSC measurements.

Considering the temperature deviations during the temperature calibration shown in Appendix A, Table A1, a range of activation energy of 226.91–244.25 kJ/mol can be determined for the non-deformed specimen and 147.82–156.24 kJ/mol for the cold-rolled specimen.

The estimated activation energy for the cold-rolled specimen in the present study is lower than the value of self-diffusion through the lattice in copper (∼197 kJ/mol) [39], whereas the activation energy for the non-deformed specimen is slightly higher. This may be due to residual scandium atoms in the copper matrix. During aging, not all scandium atoms are transferred to the Cu_4_Sc phase; residues remain in the copper matrix depending on the temperature and duration of the aging treatment. Because the alloying element has a larger atomic radius than copper, distortions in the lattice of the copper matrix result, which act as impediments to dislocation movement and thereby hinder diffusion [3]. Therefore, a higher amount of energy needs to be applied to overcome this barrier, resulting in higher activation energy.

### 3.3. Microstructure Analysis

Changes in microstructure that occur during thermal treatment can be visually investigated by metallographic analysis. An SEM backscatter detector can be used to identify differences in chemical composition and distinguish between the phases of different elemental compositions [40]. The material contrast is determined by the atomic number of the elements. As the atomic number increases, the degree of backscattering increases as well, resulting in higher brightness in the SEM image of regions containing elements with higher atomic numbers. [41].

**Figure 5:**
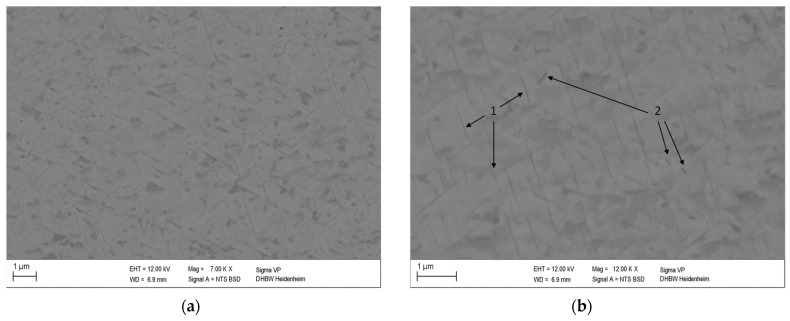
Microstructure of non-deformed CuSc0.4 specimen after DSC analysis heated up to 610 °C at a heating rate of 10 K/min with magnitude of 7000 (**a**) and 12,000 (**b**) showing homogeneously distributed lamellar Cu_4_Sc phases (1) and coarsened phases detached from the structure (2).

Figure 5 shows the microstructure of the non-deformed CuSc0.4 specimen after heating with 10 K/min up to 610 °C followed by cooling with 10 K/min. The selected temperature can be deduced from the DSC curve shown in Figure 2b, which shows that the selected temperature of 883.15 K is just below the precipitation’s final peak temperature.

Because the light metal Sc has a lower atomic number than copper [6], the Cu_4_Sc phase containing one-fifth scandium atoms appears darker compared to the copper matrix. The fine lamellar structures (dark grey) are homogeneously distributed within the copper matrix (light grey).

**Figure A2:**
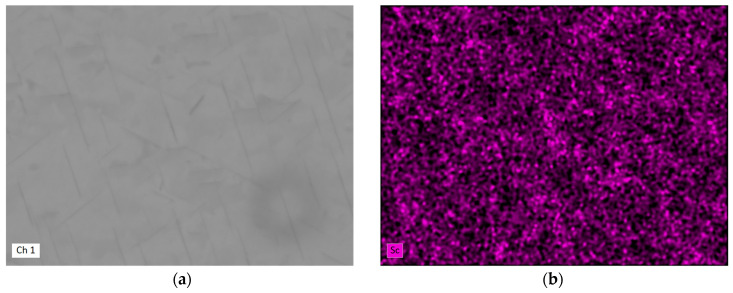
Lamellar distribution of Cu_4_Sc precipitates without prior cold deformation after DSC analysis heated up to 610 °C at a heating rate of 10 K/min, as analyzed with EDS: (**a**) backscatter detection and (**b**) EDS distribution of Sc.

The characteristics of the Cu_4_Sc precipitates are comparable to those reported by Dölling et al. [31], which were analyzed at the same heating rate and a slightly lower final temperature (600 °C). Energy Dispersive X-ray spectroscopy (EDS) proved that the darker periodically-appearing structures are directly associated with enhanced scandium content (Appendix A, Figure A2). This observation is further supported by the EDS images obtained by Dölling et al. [9,31] with a slightly lower scandium content of 0.3 wt.%.

**Figure 6:**
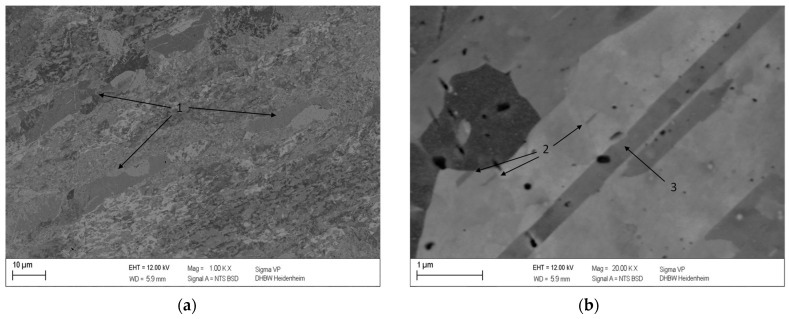
Microstructure of 75% cold-rolled CuSc0.4 specimen after DSC analysis heated up to 610 °C at a heating rate of 10 K/min with a magnitude of 1000 (**a**) and 20,000 (**b**), showing partly recrystallized areas within the strongly deformed microstructure (1), Cu_4_Sc precipitates (2), and twins (3).

In direct comparison to the microstructural images of the non-deformed specimen, the 75% cold-rolled specimen with identical heat treatment already shows the first signs of partially recrystallized areas within the microstructure (Figure 6a). This observation fits the analysis of the 10 K/min DSC curve depicted in Figure 3a. The DSC measurement shows that the precipitation reaction has already been completed at the same maximum temperature of 883.15 K and that the recrystallization of the microstructure has started. However, it is notable that the Cu_4_Sc precipitates exhibit a significant decrease in size (Figure 6b), making them discernible only at higher magnifications.

## 4. Conclusions

The present paper describes the kinetics of Cu_4_Sc precipitation of a CuSc0.4 wt.% alloy with and without prior cold working using differential scanning calorimetry (DSC). The results showed that a prior 75% cross-section reduction by cold working can accelerate the precipitation reaction and lower the reaction’s starting temperature by about 100 K. The activation energies needed for the reaction were determined by the Kissinger method and resulted in values of 226.91–244.25 kJ/mol for the non-deformed specimen and 147.82–156.24 kJ/mol for the 75% cold-rolled specimen. The differences observed in the activation energies provide new insights into the effects of cold deformation on precipitation kinetics in binary alloys, and can help in the design and optimization of such materials. The results of this study highlight the importance of carefully considering the effects of processing on precipitation behavior in order to achieve the desired material properties.

## Figures and Tables

**Table 1:** Values of maximum temperatures *T_p_* [K] of the Cu_4_Sc precipitation reaction in non-deformed (0%) and 75% cold-rolled CuSc0.4 wt.% alloy.

	*V* [K/min]	0%	75%
*Tp* [K]	5	-	711.7
	10	837.7	726.3
	15	851.4	739.7
	20	856.8	745.2
	40	872.3	769.0

**Table 2:** Mean values of estimated activation energies of the Cu_4_Sc precipitation reaction in non-deformed (0%) and cold-rolled (75%) CuSc0.4 wt.% alloy using the Kissinger method.

	0%	75%
*E* [kJ/mol]	232.02	151.51
*r* ^2^	0.9913	0.9816

## Data Availability

Not applicable.

## Appendix A

**Table A1:** Maximum deviation of the experimentally determined melting temperature [*dT*] of the calibration sample materials (In, CsCl, Ag, and Au) for heating rates of 5, 10, 15, 20, and 40 K/min.

*V* [K/min]	+*dT* [K]	−*dT* [K]
5	0.3	0.8
10	0.3	0.1
15	0.4	0.8
20	0.3	0.1
40	0.7	1.5
